# The effect of breakfast on childhood obesity: a systematic review and meta-analysis

**DOI:** 10.3389/fnut.2023.1222536

**Published:** 2023-09-06

**Authors:** Kun Wang, Yifan Niu, Zhenzhen Lu, Boyang Duo, Clement Yaw Effah, Lina Guan

**Affiliations:** ^1^Department of Anesthesiology, The First Affiliated Hospital of Zhengzhou University, Zhengzhou, Henan, China; ^2^Henan Medical School of Zhengzhou University, Zhengzhou, Henan, China; ^3^General ICU, The First Affiliated Hospital of Zhengzhou University, Zhengzhou, China

**Keywords:** breakfast, children, adolescents, obesity, overweight

## Abstract

**Objective:**

Previous cohort trials have shown that skipping breakfast increases the risk of obesity or overweight in children. However, this finding remains controversial. Through a meta-analysis, this study systematically evaluated the effect of skipping breakfast on the prevalence of obesity or overweight in children.

**Methods:**

We performed a literature search for studies published until March 19, 2023. using the Cochrane, PubMed, and Embase databases. Based on the inclusion and exclusion criteria, observational studies on the relationship between skipping breakfast and overweight/obesity in children and adolescents were analyzed. Three investigators independently screened the relevant literature, extracted the data, and assessed the risk of bias. The quality of the included studies was assessed using the Newcastle-Ottawa Scale (NOS). A random-effects model was used. The odds ratio (OR) with its 95% confidence interval (*CI*) was used to indicate the effect size.

**Results:**

A total of 40 retrospective studies with 323,244 children ranging in age from 2 to 20 years were included in this study. The results of this meta-analysis showed that children and adolescents who skipped breakfast had a significantly higher prevalence of obesity or overweight than those who ate breakfast (OR, 1.59; *95% CI*, 1.33–1.90; *P* < 0.001). Skipping breakfast was positively associated with overweight in children and adolescents (OR, 1.37; *95% CI*, 1.23–1.54; *P* < 0.001). Similarly, skipping breakfast was positively associated with obesity in children and adolescents (OR, 1.51; *95% CI*, 1.30–1.76; *P* < 0.001). The effect was also different by sex, with girls being the most affected (OR, 1.47; *95% CI*, 1.23–1.76; *P* < 0.001). There was also a correlation between skipping breakfast and abdominal obesity in children (OR, 0.65; *95% CI*, 0.55–0.77; *P* < 0.001).

**Conclusion:**

This meta-analysis suggested that skipping breakfast is associated with an increased risk of overweight/obesity in children and adolescents. The findings provide support for a possible protective role of breakfast against excessive weight gain in children and adolescents. However, more rigorous study designs with validated and standardized measures of relevant variables are needed.

## Introduction

The prevalence of obesity or overweight in children and adolescents has been increasing significantly since the 1980s. According to reports by the World Health Organization (WHO) (Global Strategy on Diet), the world obesity rate among people under 18 years of age increased from 11 million to 124 million over three decades. Although studies have confirmed that the prevalence of childhood obesity is now stabilizing and may be declining slightly in specific subgroups, it is still at extremely high levels. Children with obesity are likely to remain obese for the rest of their lives and experience a variety of other complications. The continued high prevalence of childhood obesity will place a significant burden on society. There is evidence that obese children and adolescents are at greater risk of obesity and other chronic diseases such as diabetes, cardiovascular disease, and dyslipidemia during adulthood (1–3). Therefore, the primary prevention of childhood obesity is of utmost importance. One main factor that can be ascribed to childhood obesity and overweight is skipping breakfast.

The underlying potential mechanism for this situation is still unknown. A previous study reported that skipping breakfast may be connected to lower satiety during the day and an increase in the consumption of high-fat snacks, which can be summarized as appetite control, energy intake and diet quality (4). These variations can lead to subsequent overeating and impaired insulin sensitivity. The causes of obesity are diverse. They are interconnected and affect each other in complex ways, and we cannot solve the problem of obesity with a single-sided solution. Prevention in multiple aspects of the socioecological framework is needed (5–7). The daily frequency of eating, eating occasions and times, and eating patterns can influence the digestion and absorption of calories and energy balance (8–10). Moreover, a study across 12 countries also showed that the human developmental environment, socioeconomic factors, cultural practices and the accessibility of school breakfast programs affect the frequency of eating breakfast among children (11). Regarding the latent factors of skipping breakfast in children, previous research has disclosed that there is a correlation between parents’ breakfast habits and those of their children. Children tend to skip breakfast if their parents skipped breakfast when they were 1.5 years old (12). Additionally, parental knowledge and awareness levels are also associated with children’s dietary models (13). Children have their own problems that can be exacerbated when they mimic their parents’ bad behaviors (14, 15). By precisely examining these issues, we can see that there are some differences among subgroups, such as groups classified by sex and age. Previous studies have shown that the most vulnerable group is children aged from 2 to 5 years old (16, 17). Girls are more vulnerable, because they are more likely to be exposed to an in utero environment with high sugar and undernutrition, which lead to disrupted glucose homeostasis, resulting in an increased risk of obesity and overweight (18–21). Furthermore, the cultural perspective of thinness and beauty in modern society has resulted in differences in dietary intake (22, 23).

Obviously, eating breakfast has many advantages such as improved performance in school, improved cognition (24, 25), and reductions in tardiness, absenteeism and psychosocial problems (26). Daily breakfast consumption among children and adolescents is considered an important preventive measure against obesity or overweight (27). The USDA Center for Nutrition Policy and Promotion (CNPP) conducted a systematic review (15 studies published from 2000 to 2010) on the relationship between breakfast consumption and obesity in children (28) to inform the 2010 Dietary Guidelines for Americans (DGA). The DGA committee reported that the pediatric population that skipped breakfast had an increased and moderate risk of obesity, with a stronger association found in adolescents.

The goal of this study was to provide explanations by reviewing previous studies of obesity or overweight in children and adolescent in relation to skipping breakfasts. Our research scope covered almost all related articles published in the past 25 years. Because of the large amount of extracted data, our data are more convincing and reliable. At the same time, our study involved a broader exploration of possible influencing factors, and in-depth discussions to elucidate the mechanisms that may be related to the experimental results, especially the reasons for the differences between boys and girls, as well as the mechanisms for obesity caused by skipping breakfast. We carefully compared the impact of skipping breakfast on obesity between boys and girls and on abdominal obesity in children and adolescents, which was rarely mentioned in previous meta-analyses. By understanding these differences and the underlying mechanisms, we can target relevant dietary strategies, according to different populations, physical activity levels, and types of obesity diseases, which is of great importance in improving childhood obesity.

## Methods

### Inclusion and exclusion criteria

#### Inclusion criteria

The inclusion criteria were as follows:(1) observational studies; (2) studies including children and adolescents aged 2–20 years; (3) studies including a comparison between skipping and eating breakfast in children and adolescents; (4) studies including patient outcomes of obesity, overweight, or abdominal obesity; (5) studies for which valid information could be extracted from the article, including the study duration, number of study participants, age of study participants, and outcome indicators related to the participants; (6) full text articles that could be searched and downloaded from PubMed, Embase, and the Cochrane Library; (7) studies in which the target population was children and adolescents; and (8) studies reporting either the relative risk (RR) or odds ratio (OR) and 95% confidence interval (*CI*).

#### Exclusion criteria

The exclusion of studies was performed by three researchers as follows: (1) secondary studies such as reviews and meta-analyses; (2) articles for which valid data could not be extracted or the full text could not be downloaded from the database; (3) studies that did not clearly specify the exposure factor as “skipping breakfast led to obesity”; (4) studies with non-peer-reviewed material; (5) studies including participants diagnosed with diseases relevant to the purpose of the study or hospitalized patients; and (6) studies in which overweight or obesity was considered an exposure and skipping breakfast was considered an outcome.

### Information sources

We conducted a comprehensive search in the PubMed, Embase, and Cochrane English databases for literature published before March 19, 2023. We searched for relevant studies using MeSH, Emtree, title abstracts, and keywords for the search terms “breakfast” “children,” “adolescent,” “obese” and “obesity” (shown in Supplementary Table 1).

### Literature screening

We manually screened the articles to identify those with a significant correlation between skipping breakfast and obesity or overweight in children and adolescents. First, we judged the research direction of the articles by evaluating the abstract and title. Articles that could not be judged through the abstract and title were downloaded and the full text was read. After the initial screening, three researchers jointly judged the articles that could be included and extracted the data. When there was controversy regarding whether an article should be included, one or more authoritative researchers were asked to decide.

### Data extraction

**FIGURE 1:**
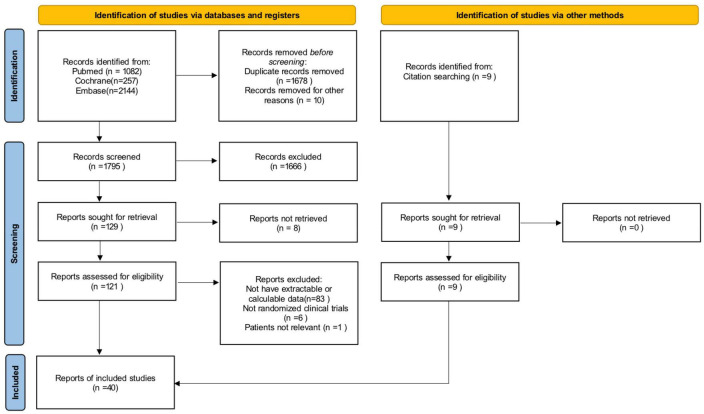
Flow diagram.

Data on the number of children with obesity, the number of children who ate breakfast and the number of children who did not eat breakfast, ORs with their 95% confidence intervals for the effect of not eating breakfast on the prevalence of obesity or overweight, as well as relevant continuous variable indicators were extracted from the included literature. A screening flow diagram was created based on the search results (Figure 1). Information such as the name of the author, the year of publication, the type of study, the sample size, age, and the main findings of the included studies are shown in Table 1. Article quality was evaluated according to the Newcastle-Ottawa Scale (NOS), as shown in Table 2.

**TABLE 1:** The basic information of the included studies.

References	Study type	Sample size	Sex	Age (yr)	Anthropometric assessment	Comparison groups	Adjustment	Key findings
Aanesen et al. (33)	Cross-section	4,360	Both sexes	5.6–7.4	Standard measurement	Skippers vs. eaters	Physical activity and commuting to school, wellbeing in school, bedtime	A statistically significant association emerged between skipping breakfast and OW/OB in girls (OR 1.66, 95% CI 1.17–2.36) but not in boys (OR 1.02, 95% CI 0.63–1.63)
Ahadi et al. (34)	Cross-section	13,486	Both sexes	6–18	Standard measurement	5–7 days a week vs. 0–2 days a week	Age and sex, family history of chronic diseases, screen time, physical activity, socio-economic status	The students who skipped breakfast had a significantly higher risk of abdominal obesity (OR 1.35, CI 95%:1.18–1.53), overweight (OR 1.16, CI 95%:1.01–1.34) and general obesity (OR 1.61, CI 95%:1.39–1.89)
Antonogeorgos et al. (36)	Cross-section	700	Both sexes	10–12	Standard measurement	Skippers vs. 0–6 days a week	Not clear	Children who consumed more than three meals per day and also consumed breakfast daily, were two times less likely to be overweight or obese (adjusted odds ratio: 0.49, 95% confidence interval: 0.27–0.88)
Andersen et al. (35)	Cross-section	1,432	Both sexes	4 grade and 8 grade	Self-reported	≤ 2 vs. 6–7 times/week eating breakfast	Age, gender, social class, watching TV and/or using computer, energy	The pupils who had breakfast 5 times or less per week had higher odds for being overweight compared with those having breakfast 6–7 times per week
Berkey et al. (37)	Longitudinal	14,586	Both sexes	9–17	Standard measurement	Skippers vs. 0–6 days a week	Age, race, menarche (girls), Tanner stage, prior BMI Z-score, physical activity, inactivity, and height growth during the year	Children who never ate breakfast were heavier (26.4% of boy never-eaters were overweight and 25.3% of girl never-eaters were overweight) than those who ate breakfast nearly every day (21.2% of boys and 15.8% of girls were overweight)
Bjornara et al. (38)	Cross-section	6,512	Both sexes	10–12	Self-reported	7 days a week vs. 0–6 days a week	Sex, ethnicity, education level	The OR of being respectively overweight or obese (compared with normal weight) was 1.2 (95 %CI 1.0, 1.4) or 1.8 (95 % CI 1.5, 2.3) for breakfast skippers
Bozic (39)	Cross-section	3,067	Both sexes	6–9	Standard measurement	7 days a week vs. 0–6 days a week	Not clear	The children who do not eat breakfast every day are more likely to be obese (OR = 1.50)
Champilomati et al. (40)	Cross-section	1,728	Both sexes	10–12	Self-reported	7 days a week vs. 0–6 days a week	Age, sex, physical activity, meals and snacks per day, annual income status, parental weight status, parental physical activity, parental educational level and parental profession.	The frequency of breakfast consumption was not associated with childhood overweight or obesity
Croezen et al. (41)	Cross-section	21,713	Both sexes	13–16	Self-reported	Skipping > 2 days vs. 0 days	Gender, family situation, ethnic background, education, smoking	In a multivariate model containing all risk factors, breakfast skipping showed the strongest relation with overweight (OR 1.68, 95% CI 1.43–1.97 for grade 2, OR 1.32 95% CI 1.14–1.54 for grade 4)
Debeila et al. (42)	Cross-section	378	Both sexes	13–20	Self-reported	Skippers vs. eaters	Ages, gender, and the number of household adults employed	Eating breakfast was associated with reduced odds of being overweight/obese (AOR = 0.6, 95% CI: 0.34–0.97)
Dialektakou and Vranas (43)	Cross-section	811	Both sexes	16	Standard measurement	Skippers vs. Regular eaters	Sex, age, ethnicity, smoking, dieting, physical activity, and parental education	A significant association between breakfast skipping and overweight or obesity was found in 35 out of the 48 logistic regression models that were used
Dubois et al. (45)	Longitudinal	1,549	Both sexes	3.7–4.7	Standard measurement	Eating breakfast every day vs. not eating breakfast every day	Sex, parental overweight/obesity, mother’s education and immigrant status, and household income	Not eating breakfast every day almost doubled the odds of being overweight at 4.5 years when child’s sex, mother’s immigrant status and number of overweight/obese parents were part of the analysis
Dubois et al. (44)	Cross-section	1,549	Both sexes	4	Standard measurement	Skippers (fewer than 7 days) vs. eaters	Sex, birth weight, mother’s immigrant status, mother’s education level, number of parents overweight/obese, mother’s smoking status, inactivity index and all included variables in the current model.	The adjusted odds of being overweight at 4 years among pre-school children was double for breakfast skippers compared with those who ate breakfast every day
Duncan (46)	Cross-section	3,397	Both sexes	7–18	Standard measurement	5 days a week vs. never	Sex, age, father education, mother education	The odds of over-weight/obesity were 0.59 times lower in participants who ate breakfast on more than 5 days in the previous week than those who did not eat breakfast
Grieger and Cobiac (49)	Cross-section	781	Boys	12–16	Self-reported	Skippers vs. eaters	Not clear	Breakfast skippers also had a higher waist circumference and BMI compared with RTEC consumers
Dupuy et al. (29)	Cross-section	7,154	Both sexes	11–15	Self-reported	Not daily eating vs. Daily eating	Age, sex, marital status, educational level, monthly income, cigarette smoking, alcohol consumption, betel quid chewing and exercise habit	The adjusted odds ratios for the association with overweight were 0.73 (0.60–0.88) for eating breakfast daily
Fayet-moore et al. (47)	Cross-section	4,487	Both sexes	2–16	Standard measurement	Skippers vs. eaters	Age, gender, total daily energy intake and physical activity level	The prevalence of overweight was lower among breakfast consumers compared to skippers, and among cereal consumers compared to-cereal consumers (*p* < 0.001)
Lopez-Gil et al. (48)	Cross-section	2,890	Both sexes	6–7	Self-reported	Skippers vs. eaters	Age, region, and socioeconomic status	Skipping breakfast was associated with higher odds of having excess weight in boys (OR = 1.83; CI 95%, 1.18–2.86) and girls (OR = 1.98; CI 95%, 1.27–3.07)
Harding et al. (50)	Cross-section	6,599	Both sexes	11–13	Standard measurement	Skippers vs. eaters	Socio-economic circumstances, family type, height (SD), pubertal stage and age	Skipping breakfast [girls OR 1.74, CI 95% 1.30–2.34; boys OR 2.06; CI 1.57–2.70]
Haines et al. (51)	Longitudinal	2,516	Both sexes	12–17	Self-reported	Skippers vs. eaters	Age cohort, socioeconomic status, and race/ethnicity.	Greater frequency of breakfast consumption at 1998 to 1999 was protective against overweight
Khan et al. (52)	Cross-section	793	Both sexes	12–17	Standard measured	5–7 days a week vs. 0–4 days a week	Gender, age, walking to school, involvement in non-team sports at school and family income	Participants who missed breakfast had a 2.6 times higher odds of being obese than those who regularly ate breakfast (OR 2.62) with a 95% confidence interval (95% CI) of 1.35–5.08 (*p* = 0.005)
Kim and So (30)	Longitudinal	72,399	Both sexes	15	Self-reported	1–2 times/weeks vs. 6–7 times/weeks	Age, frequency of smoking, frequency of drinking, the parents’ education level, economic status, frequency of vigorous physical activity (PA), frequency of moderate PA, frequency of muscular strength exercises, mental stress, and sleep duration	The frequency of breakfast eating has no correlation with overweight/obesity in Korean adolescents
Kupers et al. (53)	Longitudinal	1,366	Both sexes	2 or 5	Standard measured	7 days a week vs. 0–6 days a week	Gender, exact age at 2 and 5 years, birth weight, origin (Dutch or non-Dutch), maternal educational level, maternal and paternal BMI at 2 or 5 years and family type	Skipping breakfast was not associated with adiposity status cross-sectionally at 2 or 5 years of age, or over time (OR: 0.72, CI: 0.15, 3.49).
Maddah (2008)	Cross-section	2,090	Girls	14–17	Standard measured	Skippers vs. eaters	Maternal educational level, TV viewing, walking	In urban residents, skipping breakfast OR = 1.96 (1.52–2.35) were independently related to overweight and obesity. In rural residents, skipping breakfast OR = 2.23 (1.37–3.65) were independently related to overweight and obesity.
Maddah and Nikooyeh (54)	Cross-section	6,635	Both sexes	6–11	Standard measured	Skipping vs. non-skipping (no clear definition)	Age, sex, maternal educational level, television viewing, birth rank, mother’s employment, parental overweight/obesity, walking, birth weight	Skipping breakfast (OR = 1.4, 95% CI, 1.2–1.7, *p* < 0.01)
Maddah and Nikooyeh (31)	Cross-section	2,577	Girls	12–17	Self-reported	Regular vs. not regular	Age and birth weight	Prevalence of over-weight was significantly higher in those who usually skipped breakfast than those who usually had their breakfast in the home
Thompson-McCormick et al. (32)	Cross-section	523	Girls	15–20	Standard measurement	Skipped breakfast at least once per week vs. ate breakfast everyday	Unadjusted	More frequent breakfast skipping was associated with greater odds of overweight [OR = 1.15, confidence interval (CI) = 1.06, 1.26, *p* < 0.01] and obesity (OR = 1.18, CI = 1.05, 1.33, *p* < 0.01)
Merten et al. (56)	Longitudinal	7,788	Both sexes	12–19	Self-reported	4–7 days a week vs. 0–3 days a week	Not clear	Breakfast consumption in adolescence significantly decreased the likelihood of chronic obesity (OR = 0.59; 95% CI: 0.52–0.68)
Mihrshahi et al. (57)	Cross-section	7,555	Both sexes	5–16	Standard measurement	Skippers vs. eaters	age, gender, socio-economic status, rural/urban residence and physical activity	Both children and adolescents who did not consume breakfast daily were more likely to be overweight/obese OR (95% CI) = 1.39 (1.07–1.81), OR (95% CI) = 1.42 (1.16–1.74)
Nilsen et al. (58)	Cross-section	2,620	Both sexes	7–9	Standard measurement	Skipper vs. eaters	Gender, parents’ weight status education and area of residence	The odds of being OW/OB was higher among those not having breakfast every day [OR 1.9, 95% confidence interval (CI) 1.20–2.96]
Ober et al. (59)	Cross-section	1,215	Both sexes	Grade 4 and grades 6–8	Standard measurement	Skipper vs. eaters	Age, socio–economic status, school type, migration background, and parental weight status	Having breakfast was associated with a lower risk of being overweight (ORadj = 0.30, *p* = 0.009), while having two breakfasts resulting in stronger associations (risk of overweight: ORadj = 0.22, *p* = 0.001)
Okada et al. (12)	Longitudinal	42,663	Both sexes	2.5–12	Self-reported	Skipper vs. eaters	Sex, birth weight, feeding status, hospital admission history, and parental education and working status	When compared to children who did no skip breakfast, those who skipped breakfast had 18–116% increased risk of childhood overweight/obesity; ORs were 1.18 (95% CI 1.05–1.32) and 2.16 (95% CI 1.55–2.99), respectively
Olson et al. (60)	Cross-section	105	Both sexes	10.79 (mean)	Standard measurement	Skipper vs. eaters	Urban, age, sex	Frequency of breakfast consumed weekly (OR = 0.699, *p* < 0.01) were significant predictors of being overweight or obese
Panagiotakos et al. (61)	Cross-section	700	Both sexes	10–12	Standard measurement	Skipper vs. eaters	Physical activity and other potential confounders	The odds ratio of overweight/obesity for boys who ate daily breakfast was 0.51 (95% CI: 0.25–1.05), and for girls was 0.27 (95% CI: 0.12–0.64)
Sandercock et al. (62)	Cross-section	4,326	Both sexes	10–16	Standard measurement	5 days a week vs. 0–4 days a week	Not clear	Participants who sometimes ate breakfast were more likely to be obese than those who always did (*P* < 0.05)
Shafiee et al. (63)	Cross-section	5,604	Both sexes	10–18	Standard measurement	0–2 days/weeks vs. 6–7 days/weeks	Age, sex, family history of chronic disease, educations of parents, physical activity	Seldom breakfast eaters had an increased risk of obesity compared to “regular breakfast eaters”
Vik et al. (65)	Cross-section	7,716	Both sexes	11.5 ± 0.7	Standard measurement	5–7 days a week vs. 2–4 days a week	Gender, ethnicity, parental education and country	Having regular family breakfast, but not lunch or dinner, was inversely associated with overweight [OR = 0.78 (95% CI 0.67–0.91)]
Yaguchi-Tanaka and Tabuchi (16)	longitudinal	53,575	Both sexes	2.5–13	Self-reported	Skipper vs. eaters	Birth weight, breastfeeding exclusiveness, maternal age, paternal age, maternal educational level, and paternal educational level, hours spent watching television, hours spent playing computer games (exclude 2.5 and 13 years old) and living with a grandparent, at each analytical age	Skipping breakfast at 2.5 years old was significantly associated with overweight/obesity at 7 years old in boys (OR 1.21; 95% CI, 1.03–1.43), but not in girls (OR 1.24; 95% CI, 1.06–1.47)
Traub et al. (64)	Longitudinal	1,733	Both sexes	7.08 ± 0.6	Standard measurement	Skipper vs. eaters	School, migration background, family education level, household income, age, gender, participation in the intervention	Skipping breakfast led to increased changes in WHtR, weight and BMI measures
Wadolowska et al. (66)	Cross-section	1,566	Both sexes	11–13	Standard measurement	Skipper vs. 0–3 days a week	Gender, age, residence, Family Affluence Scale, nutrition knowledge, physical activity, screen time	In comparison to “never-skippers,” “frequent breakfast skippers” were more likely to be overweight/obese (odds ratio, OR 1.89; 95% confidence interval, 95% CI 1.38, 2.58)

CI, confidence interval; OR, odd ratio.

**TABLE 2:** The Newcastle-Ottawa quality assessment scale of including studies.

References	Selection				Comparability		Assessment of outcome			Total quality score
**First author**	**Representativeness of exposure arm(s)**	**Selection of the comparative arm(s)**	**Origin of exposure source**	**Demonstration that outcome of interest was not present at start of study**	**Studies controlling the most important factors**	**Studies controlling the other main factors**	**Assessment of outcome with independency**	**Adequacy of follow-up length (to assess outcome)**	**Lost to follow-up acceptable (less than 10% and reported)**	
Aanesen et al. (33)	*	*	*	–	*	*	*	*	–	7
Ahadi et al. (34)	*	*	*	*	*	*	*	*	–	8
Andersen et al. (35)	*	*	–	*	*	*	*	*	–	7
Antonogeorgos et al. (36)	*	*	*	–	*	–	*	*	–	6
Berkey et al., (37)	*	*	*	–	*	*	*	*	–	7
Bjornara et al. (38)	*	*	–	–	*	*	*	*	–	6
Bozic et al. (39)	*	*	*	–	*	–	*	*	–	6
Champilomati et al. (40)	*	*	–	–	*	*	*	*	–	6
Croezen et al. (41)	*	*	–	–	*	*	*	*	–	6
Debeila et al. (42)	*	*	–	–	*	*	*	*	–	6
Dialektakou and Vranas (43)	*	*	*	–	*	*	*	*	–	7
Dubois et al. (45)	*	*	*	*	*	*	*	*	–	8
Dubois et al. (44)	*	*	*	–	*	*	*	*	–	7
Duncan (46)	*	*	*	–	*	*	*	*	–	7
Dupuy et al. (29)	*	*	*	–	*	*	*	*	–	7
Fayet-moore et al. (47)	*	*	*	*	*	*	*	*	–	8
Lopez-Gil et al. (48)	*	*	–	*	*	*	*	*	–	7
Grieger and Cobiac (49)	*	*	–	–	*	*	*	*	–	6
Harding et al. (50)	*	*	*	–	*	*	*	*	–	7
Haines et al. (51)	*	*	–	–	*	*	*	*	–	6
Khan et al. (52)	*	*	*	*	*	*	*	*	–	8
Kim and So (30)	*	*	–	–	*	*	*	*	–	6
Kupers et al. (53)	*	*	*	*	*	*	*	*	–	8
Maddah (2008)	*	*	*	–	*	*	*	*	–	7
Maddah and Nikooyeh (54)	*	*	*	–	*	*	*	*	–	7
Maddah and Nikooyeh (31)	*	*	–	–	*	*	*	*	–	6
Thompson-McCormick et al. (32)	*	*	*	*	*	–	*	*	–	7
Merten et al. (56)	*	*	–	–	*	–	*	*	–	5
Mihrshahi et al. (57)	*	*	*	–	*	*	*	*	–	7
Nilsen et al. (58)	*	*	*	–	*	*	*	*	–	7
Ober et al. (59)	*	*	*	*	*	*	*	*	–	8
Okada et al. (12)	*	*	*	*	*	*	*	*	–	8
Olson et al. (60)	*	*	*	–	*	*	*	*	–	7
Panagiotakos et al. (61)	*	*	*	–	*	*	*	*	–	7
Sandercock et al. (62)	*	*	*	–	*	–	*	*	–	6
Shafiee et al. (63)	*	*	*	–	*	*	*	*	–	7
Yaguchi-Tanaka and Tabuchi (16)	*	*	–	*	*	*	*	*	–	7
Traub et al. (64)	*	*	*	–	*	*	*	*	–	7
Vik et al. (65)	*	*	*	–	*	*	*	*	–	7
Wadolowska et al. (66)	*	*	*	*	*	*	*	*	–	8

*Means scoring one point in total quality score.

### Statistical analysis

All statistical analyses were conducted using STATA12.0. Our statistics were pooled with the random-effects model. This model was deemed more appropriate than the fixed-effects model because the studies included in this meta-analysis represented samples from different populations. Studies that reported rate ratios were converted to ORs using the methods defined in the Cochrane Handbook for Systematic Reviews of Interventions. Summary statistics are expressed as ORs and 95% CIs. Statistical heterogeneity was assessed using the I^2^ statistic. Publication bias was assessed qualitatively by visual inspection of the inverted funnel. Egger’s test was performed to assess small study effects. Statistical tests were two-tailed, and the statistical significance threshold was *P* < 0.05. For cross-sectional studies, sensitivity analyses based on funnel plots and Egger tests were used to show the robustness of the results and to confirm the presence of publication bias. To check the robustness of the results, a sensitivity analysis was performed. A funnel plot can visually represent a graph as an inverted funnel with good symmetry, which indicates less bias. To test the relationship between skipping breakfast and the risk of obesity, the pooled RR and *95% CI* for skipping breakfast versus not skipping breakfast were calculated using a random effects model. Furthermore, we used the I^2^ test to assess the heterogeneity of the included studies; an I^2^ value greater than 50% was considered to indicate substantial heterogeneity. To determine the source of the heterogeneity among the included studies, subgroup analyses were performed if there were at least 2 studies in each category. Egger regression asymmetry tests were used. Egger regression and funnel plots used to evaluate publication bias.

## Results

### Literature search results

Three researchers used the PubMed, Embase, and Cochrane Library electronic databases to search for literature based on the search terms “child,” “children,” “adolescent,” “breakfast,” “obese,” and “obesity.” There were 3491 articles, including 1082 articles retrieved from PubMed, 2,144 articles retrieved from Embase, and 257 articles retrieved from the Cochrane Library. After the manual screening of titles and abstracts, 129 articles were initially included. Of these, 83 articles did not have extractable or calculable data. One article had an experimental population other than children and adolescents, and 6 papers were secondary studies such as reviews and meta-analyses. After excluding all studies that did not meet the inclusion criteria, 40 studies were ultimately retained and included in this study. The flow chart of the literature screening process is shown in Figure 1.

### Basic characteristics of the included literature

A total of 40 studies including 8 longitudinal studies and 32 cross-sectional studies, were included in this meta-analysis (12, 16, 29–66). The total number of patients included in the 40 studies was 323,244. The researchers extracted data related to author information, the year of publication, the study type, the sample size, age, and main findings from the 40 studies to determine the basic characteristics of the articles (Table 1).

### Quality evaluation of included literature

In this meta-analysis, 3 reviewers independently assessed the quality of the 40 articles by using the Newcastle-Ottawa Scale (NOS). The objective assessments for the following dimensions yielded a maximum score of 9; study population selection (no more than 4 points), comparability of cohort design and analysis (no more than 2 points), and adequacy of outcome measures (no more than 3 points). Score of 7–9 were considered to indicate high quality (low risk of bias). One article had a score of 5, and the remaining articles had scores ≥ 6. Eight of the included articles had a quality assessment score of 8. Twenty articles had a score of 7, and 11 articles had a score of 6, indicating a low risk of bias based on the NOS. The articles were of high quality, were designed in a reasonable manner, had reliable data, and could be included in the meta-analysis. The results of the quality assessment are shown in Table 2.

### Outcomes

**FIGURE 2:**
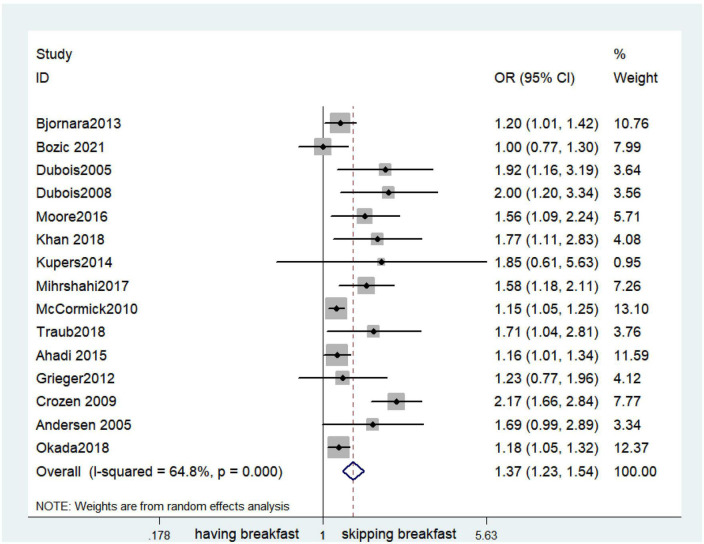
Skipping breakfast was positively associated with overweight.

**FIGURE 3:**
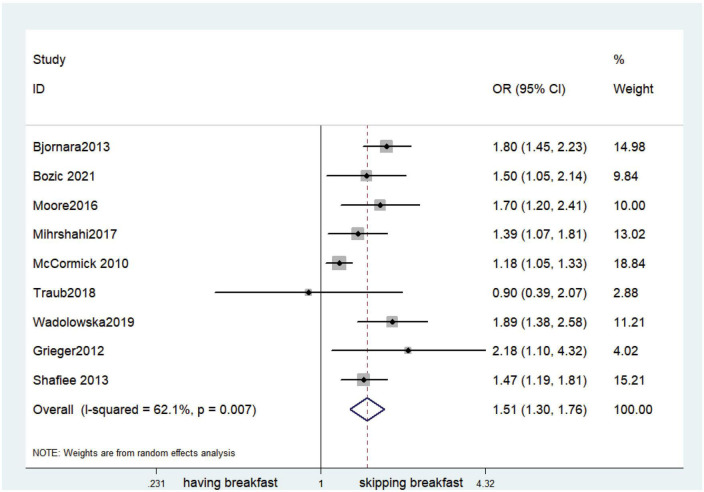
Skipping breakfast was positively associated with obesity.

**FIGURE 4:**
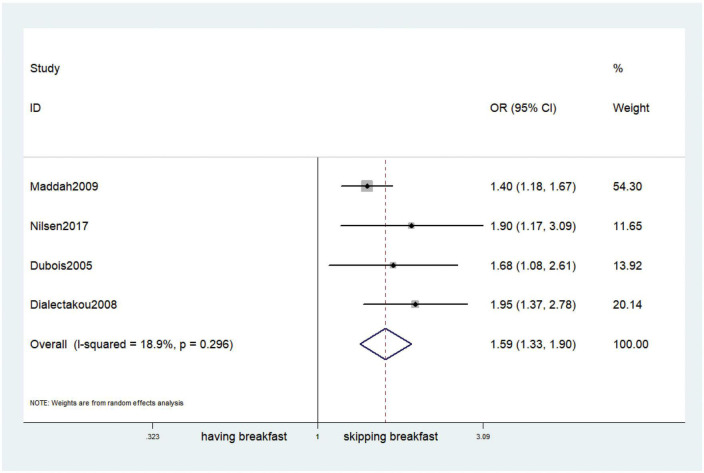
Skipping breakfast on overweight or obesity.

**FIGURE 5:**
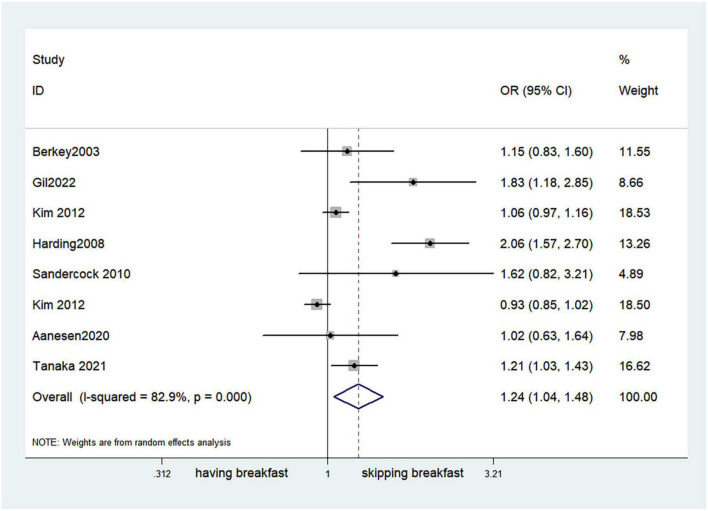
Skipping breakfast and the risk of obesity in boys.

**FIGURE 6:**
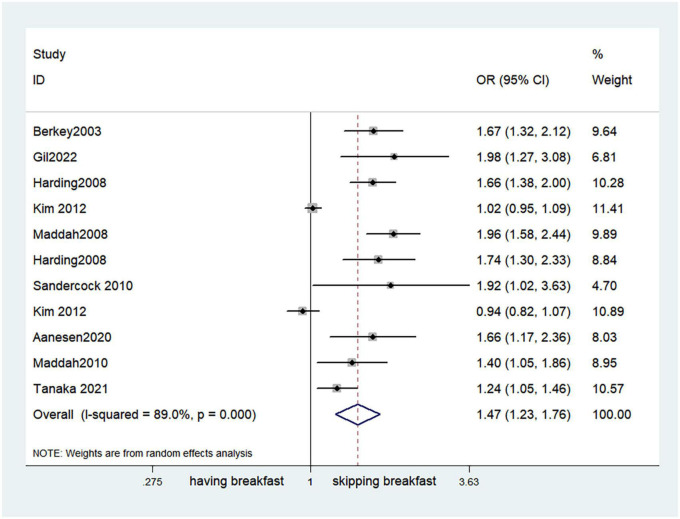
Skipping breakfast and the risk of obesity in girls.

**FIGURE 7:**
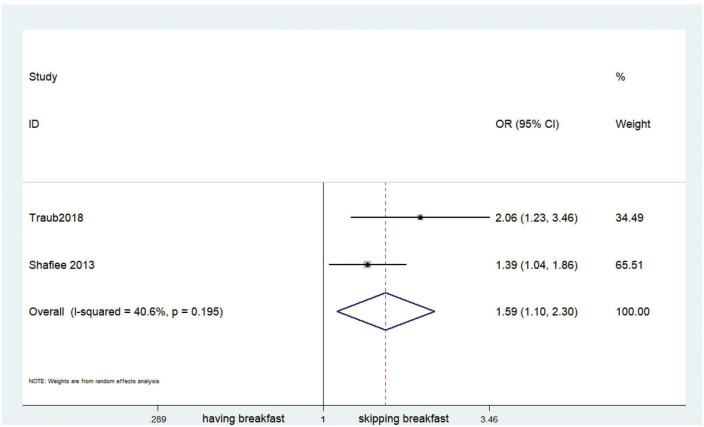
Skipping breakfast and abdominal obesity.

**FIGURE 8:**
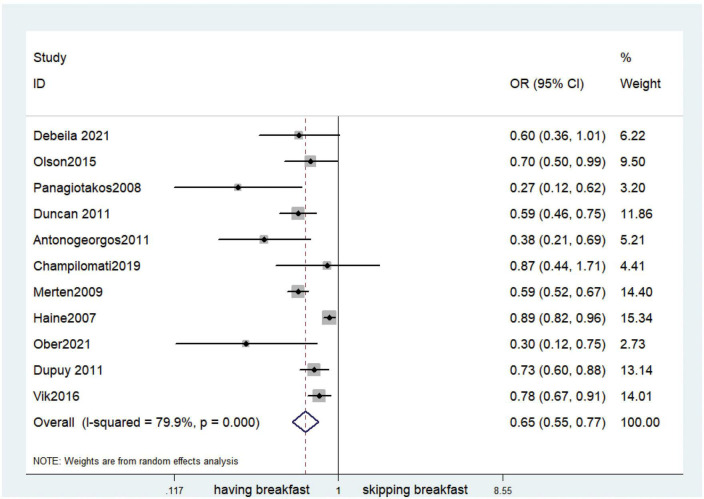
Eating breakfast on overweight or obesity.

Fifteen observational studies (12, 32, 34, 35, 38, 39, 41, 44, 45, 47, 49, 52, 53, 57, 64) showed that skipping breakfast was positively associated with overweight in children and adolescents (OR, 1.37; 95% *CI*, 1.23–1.54; *P* < 0.001; Figure 2). Nine studies (32, 38, 39, 47, 49, 57, 63, 64, 66) illustrated that skipping breakfast was positively associated with obesity in children and adolescents (OR, 1.51; 95% *CI*, 1.30–1.76; *P* < 0.001; Figure 3). Four articles (43, 45, 54, 58) provided data on the effect of skipping breakfast on overweight or obesity in children and adolescents (OR, 1.59; 95% *CI*, 1.33–1.90; *P* < 0.001; Figure 4). In the cross-sectional study, the subgroup analysis of sex showed 19 effect values extracted from 10 observational studies (16, 30, 31, 33, 37, 48, 50, 54, 62), and the results showed that skipping breakfast had different effects on obesity in boys and girls. Subgroup analysis of the cross-sectional studies showed an association between skipping breakfast and the risk of obesity in boys (OR, 1.24; 95% *CI*, 1.04–1.48; *P* = 0.014; Figure 5) and girls (OR, 1.47; 95% *CI*, 1.23–1.76; *P* < 0.001; Figure 6). The analysis by sex was performed because girls are more likely to skip breakfast than boys, as well as to determine the differential impact of breakfast on boys and girls. There is literature indicating that skipping breakfast has a statistically significant effect on obesity in girls, while the correlation between skipping breakfast and obesity in boys is not statistically significant (33). One study indicated that skipping breakfast was not associated with childhood obesity (40). We analyzed abdominal obesity in children, and 3 articles (63, 64, 67) reported the effect on abdominal obesity; 2 (63, 64) mentioned the effect of skipping breakfast on abdominal obesity, and 1 (67) mentioned that eating breakfast reduced the risk of abdominal obesity. Forest plotting was performed using the data from the two articles on the effect of skipping breakfast on abdominal obesity and showed a positive association between skipping breakfast and abdominal obesity (OR, 1.59; 95% *CI*, 1.10–2.30; *P* = 0.013; Figure 7). Because only two articles were included, the heterogeneity was high (*P* = 0.195). In addition, the age, sex, living environment, family atmosphere and other factors of the respondents will had an impact on the survey, creating heterogeneity among the study results. There were also 11 articles (29, 36, 40, 42, 46, 51, 56, 59–61, 65) that described the effect of eating breakfast on overweight or obesity risk in children and adolescents (OR, 0.65; 95% *CI*, 0.55-.77; *P* < 0.001; Figure 8).

### Sensitivity analysis and publication bias

**FIGURE 9:**
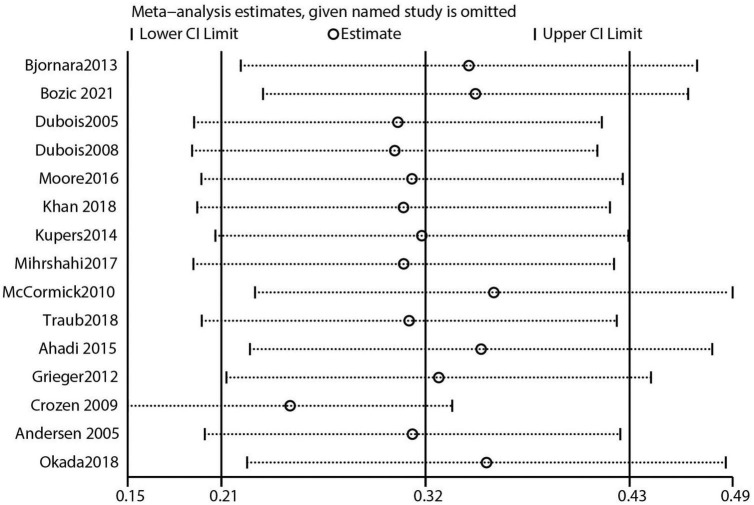
Skipping breakfast was positively associated with overweight.

The 40 included studies were mainly those with high quality assessment scores (NOS scores). After performing leave-one-out sensitivity analysis, combined effect sizes, and all effect sizes were included in the total 95% *CI* effect size, indicating good stability of the results. The data results are shown in Figure 9 and Supplementary Figures 1–4. The funnel plot visually represents the graph as an inverted funnel with good symmetry, indicating less bias. The results of data analysis are shown in Supplementary Figures 5–9. According to the results of the Egger test, studies that reported the effect of skipping breakfast on overweight risk in children had some publication bias (Supplementary Figure 10; *P* = 0.004). However, studies that reported the effect of skipping breakfast on obesity risk had no major bias (Supplementary Figure 11; *P* = 0.060). Studies that reported the effect of eating breakfast on obesity or overweigh risk had some bias (Supplementary Figure 12; *P* = 0.023). Additionally, there was some publication bias among studies that reported the effect of skipping breakfast on childhood obesity or overweight risk in boys (Supplementary Figure 13; *P* = 0.062), but no major publication bias was observed in studies that reported the same in girls (Supplementary Figure 14; *P* = 0.003).

## Discussion

In this study, we summarized 40 high-quality articles on the effect of skipping breakfast on OW/OB risk in children and adolescents. In the screening process, we excluded secondary research articles such as review articles and meta-analyses, extracted data from 40 articles, and used STATA 12.0 software to perform various analyses and to draw different graphs. In this study, we addressed whether skipping breakfast causes OW/OB in children and adolescents, and whether skipping breakfast causes different results in different sexes (male and female children and adolescents). The results were that skipping breakfast increased the prevalence of OW/OB (OR, 1.59; *95% CI*, 1.33–1.90; *P* < 0.001; Figure 4) in children and adolescents. Most articles in this meta-analysis were cross-sectional studies. However, cross-sectional studies cannot make judgment about causality. Eight other cohort studies on such issues were included in our study, and the results were consistent with our hypothesis. These results are consistent with those of previous cross-sectional studies and several small prospective cohort studies conducted outside Europe (45, 68). There is a significant association between skipping breakfast and OW/OB in children and adolescents (69, 70). The relationship between skipping breakfast and obesity or overweight risk in children and adolescents is complicated by a number of factors. Confounding factors are a major threat in observational studies, and in the course of studying the effects of skipping breakfast on OW/OB risk in children and adolescents, possible influencing factors may include socioeconomic status, region, smoking, watching TV, etc. In previous studies (30, 35, 51, 59), these factors were apparently related to obesity. For socioeconomic status, the reason may be that children in areas with poor economic conditions are more likely to skip breakfast, thus leading to obesity (59). For smoking, people who smoke generally have poor lifestyle habits and are more likely to skip breakfast, which leads to obesity. Children who like to watch TV and use computers for long periods of time are often sedentary, lack of activity and exercise, and are more likely to develop obesity (35).

Environments may differ from each other. Human development levels, social factors, schools providing breakfast, and cultural differences may lead to different prevalence rates. In a study of children aged 9–16 years in twelve countries around the world, significant differences were found in Australia, Finland, and Kenya. Horikawa et al. found that skipping breakfast led to a 75% increased risk of OW/OB in children and adolescents in the Asia-Pacific region and the prevalence may be higher in rural children than in urban children (27).

In our screening, there appeared to be a stronger association between eating or not eating breakfast and the risk of obesity and overweight in girls (OR, 1.47; *95% CI*, 1.23–1.76; *P* < 0.001; Figure 6) (30, 31, 48, 50, 55). Women are more likely to be exposed to high-glucose environments, and the presence of a uterus may be associated with the disruption of homeostasis by glucose and increased adiposity (33). In a previous study (71), we identified four different neuronal populations with respect to glucose sensitivity: the non-glucose sensing (NGS), glucose-exciting (GE), non-adapting glucose-inhibited (GI) and adapting glucose-inhibited (AdGI) populations. When extracellular glucose decreased, the activity of GE, non-adaptive GI, and AdGI neurons changed. More importantly that the relative percentage of glucose-sensitive subtypes (AdGI, non-adaptive GI, GE, and NGS neurons) within the VL-VMN was sexually dimorphic. In women, AdGI neurons and non-adapting GI neurons occur at approximately the same proportion, while in men, AdGI neurons occur approximately half as often as non-adapting GI neurons. When glucose decreased, the degree of depolarization of male and female non-adapting GI neurons was similar, but males had 2-fold increased IR due to decreased glucose compared to female, indicating increased excitability of these neurons under low-glucose conditions, which makes females more likely to develop obesity than males (71). Moreover, depending on cultural perception, girls may choose to eat less, which also includes skipping breakfast, mainly to maintain a thin body as a beauty standard (55). Middle school girls who skip breakfast are more likely to report lower levels of physical activity than those who eat breakfast regularly, which may be a contributing factor. In addition, girls with overweight and obesity may avoid exercise because they are more self-aware of their bodies (72). The development of breakfast habits in children is closely related to the habits of parents. In a Japanese study that included 42,663 children, 12–32% of their parents skipped breakfast by the time the children were one and a half years old, and this habit was mimicked by their children (27).

Children who eat breakfast differ significantly from those who do not eat breakfast in terms of diet composition. Children who do not eat breakfast have elevated daily energy fat intake, along with a reduced intake of protein, vitamins and minerals, and a stronger appetite for fat, such as fat-rich snacks, which leads to higher total plasma cholesterol levels. Additionally, the intake of breakfast cereals seems to play an important role. A study on adults found that those who ate cooked breakfast cereals had a significantly lower BMI than those who ate meat, eggs, dairy products, etc., for breakfast (73). Other studies found that there is a high prevalence of overweight among people who did not eat breakfast cereals compared to those who did (74, 75).

The mechanism of breakfast regulation in the development of obesity is very complex, and thus far, there is no clear mechanism to elucidate the relationship between them. In reviewing previous studies, we found that the possible mechanisms are mainly as follows:

Eating breakfast regularly may result in greater metabolic function, and a fiber rich breakfast may improve postprandial glycemic response, satiety, and insulin sensitivity (18). Second, skipping breakfast increases total energy intake. In a weight loss intervention trial with adolescents aged 11–16 years, it was found that an increase in energy consumption during breakfast led to a decrease in total energy intake, with an increase in the total energy intake of 171 kcal/day (76). Additionally, children who eat breakfast regularly have enhanced activity, which is considered one of the reasons for the causal relationship between skipping breakfast and OW/OB risk in children and adolescents. A recent study conducted by Professor Daniela Jakubowicz elaborated on the metabolic mechanism. His team clarified that skipping breakfast has an important connection with the clock gene that could regulate the glucose level, called the insulin response, a reaction that could lower iGLP-1 and damage the insulin response. Skipping breakfast was associated with a significantly higher post lunch and post dinner glycemic response than eating breakfast. Skipping breakfast can also lead to impaired insulin secretion after lunch and dinner, manifested by delayed insulin peaks and decreased plasma insulin and C-peptide concentrations. In addition, after lunch and dinner, the iGLP-1 response to skipping breakfast was lower than the iGLP-1 response to eating breakfast. In contrast, plasma free fatty acid and glucagon levels were significantly elevated after lunch and dinner in response to skipping breakfast. Therefore, breakfast is essential for glucose homeostasis throughout the day, including islet and incretin hormone function (77). Skipping breakfast also leads to a reduction in the expressions of gene, such as Per1, Cry1, Ror, Sirt1, and Clock, which regulate the secretion of circadian hormones and postprandial glycaemia (78). A relevant clinical trial also showed an association between overactivity in the hypothalamic-pituitary-adrenal (HPA) axis, leading to disordered cortisol rhythms (79).

The possible mechanism of abdominal obesity remains uncertain. A previous study (63) reported that people who rarely ate breakfast had lower total energy intake, but a higher body mass index than those who ate breakfast. People who typically eat breakfast consume more energy, carbohydrates, and fiber, and fat accounts for a lower percentage of the total calories; Additionally, people who eat breakfast every day appear to be more active than those who do not. This diet has the potential to improve energy balance and possibly improve glucose and insulin parameters, leading to increased satiety and a lower prevalence of abdominal obesity.

The 40 articles included retrospective cohort studies and retrospective observational studies, which had a positive effect in demonstrating that skipping breakfast can increase the prevalence of OW/OB in children and adolescents, and can provide a theoretical basis for the development healthy eating strategies and policies. We screened a large number of studies containing keywords such as children, breakfast, and obesity. We gradually censored and filtered them to ultimately obtain 40 articles that fit the study topic, and the results obtained were more objective and precise. The sample size of each of the 40 articles was very large, and the obtained results were also highly credible. Finally, the sensitivity analysis indicated that the stability of the data results was good.

The limitations are that most of the included studies were cross-sectional studies that had limitations such as lack of causality and generalization. Second, some studies were not found, and we mostly chose to exclude papers published in local languages. We only included published reports and did not search for unpublished articles. Furthermore, some studies were very old, and there may be a slight gap between previous research results and current developments. Another potential limitation arises from the various definitions of skipping breakfast. The criteria for skipping breakfast in the trials included in this review varied from study to study and sometimes did not allow for direct comparisons.

## Conclusion

The findings provide support for a possible positive role of breakfast in preventing excessive obesity in children and adolescents. However, drawing causal conclusions from the collective evidence is limited by methodological constraints and inconsistencies, including the study design, follow-up duration and frequency, exposure and outcome assessment, and limited consideration of confounding, mediating, and modifying variables. More rigorous study designs with validated and standardized measures of relevant variables are needed. Second, the mechanisms regarding the modulation of obesity by eating breakfast remain unclear and need to be further explored.

## Data availability statement

The original contributions presented in this study are included in the article/Supplementary material, further inquiries can be directed to the corresponding author.

## Author contributions

KW, YN, ZL, and BD designed the ideas for this study. YN, ZL, BD, CE, and LG performed all the procedures and data analysis. KW wrote the manuscript. All authors contributed to the article and approved the submitted version.
