# Supplementary material for: The effect of breakfast on childhood obesity: a systematic review and meta-analysis
Source: Front Nutr. 2023 Sep 6;10:1222536. doi: 10.3389/fnut.2023.1222536 (PMC10510410; doi:10.3389/fnut.2023.1222536)
Supplement: Supplementary file 1 [file Table_1.DOCX]

| **PubMed** |  |  |
| --- | --- | --- |
| 1 | (((((((child[MeSH Terms]) OR (child[Title/Abstract])) OR (children[Title/Abstract])) OR (children[MeSH Terms])) OR (adolescent[MeSH Terms])) OR (adolescent[Title/Abstract])) AND ((((obese[Title/Abstract]) OR (obesity[Title/Abstract])) OR (obese[MeSH Terms])) OR (obesity[MeSH Terms])) OR (obesity[MeSH Terms ))) AND ((fast[MeSH Terms]) OR (fast[Title/Abstract])) | **1082** |
| **Cochrane** |  |  |
| 1 | MeSH descriptor: [Child] this term only | 62191 |
| 2 | (child):ti,ab,kw (Word variations have been searched) | 173095 |
| 3 | (fast):ti,ab,kw (Word variations have been searched) | 8208 |
| 4 | MeSH descriptor: [Breakfast] this term only | 422 |
| 5 | MeSH descriptor: [Obesity] this term only | 14824 |
| 6 | (obesity):ti,ab,kw (Word variations have been searched) | 51602 |
| 7 | MeSH descriptor: [Adolescent] this term only | 121545 |
| 8 | (adolescent):ti,ab,kw (Word variations have been searched) | 157445 |
| 9 | #1 or #2 | 173095 |
| 10 | #7 or #8 | 157445 |
| 11 | #9 or #10 | 275482 |
| 12 | #5 or #6 | 51602 |
| 13 | #3 or #4 | 8208 |
| 14 | #11 and #12 and #13 | **257** |
| **Embase** |  |  |
| 1 | 'obesity'/de | 526028 |
| 2 | 'obesity':ab,ti | 438775 |
| 3 | 'fat':ab,ti | 413034 |
| 4 | 'child'/de | 2241009 |
| 5 | 'adolescent':ab,ti | 190436 |
| 6 | 'child':ab,ti | 546848 |
| 7 | 'breakfast'/de | 21781 |
| 8 | 'breakfast':ab,ti | 16505 |
| 9 | #1 OR #2 OR #3 | 950458 |
| 10 | #4 OR #5 OR #6 | 2586233 |
| 11 | #7 OR #8 | 30528 |
| 12 | #9 AND #10 AND #11 | **2144** |

**STable 1. The detailed search strategy.**
